# Frequent users of emergency services: associated factors and reasons for seeking care^1^

**DOI:** 10.1590/0104-1169.0072.2560

**Published:** 2015

**Authors:** Aline Marques Acosta, Maria Alice Dias da Silva Lima

**Affiliations:** 2 Doctoral student, Escola de Enfermagem, Universidade Federal do Rio Grande do Sul, Porto Alegre, RS, Brazil. Scholarship holder from Coordenação de Aperfeiçoamento de Pessoal de Nível Superior (CAPES), Brazil; 3 PhD, Associate Professor, Escola de Enfermagem, Universidade Federal do Rio Grande do Sul, Porto Alegre, RS, Brazil

**Keywords:** Emergency Services, Hospital, Emergency Nursing, Health Service Needs and Demands

## Abstract

**Aim:**

to identify the profile of frequent users of emergency services, to verify the associated factors and to analyze the reasons for the frequent use of the services.

**METHOD:**

An explanatory sequential type mixed method was adopted. Quantitative data were collected from the electronic medical records, with a sample of 385 users attended four or more times in an emergency service, during the year 2011. Qualitative data were collected through semi-structured interviews with 18 users, intentionally selected from the results of the quantitative stage. Quantitative data were analyzed using descriptive and inferential statistics and qualitative data using thematic analysis.

**RESULTS:**

It was found that 42.9% were elderly, 84.9% had chronic diseases, 63.5% were classified as urgent, 42.1% stayed for more than 24 hours in the service and 46.5% were discharged. Scheduled follow-up appointment, risk classification, length of stay and outcome were factors associated with frequent use. The reasons for seeking the services were mainly related to the exacerbation of chronic diseases, to easier access and concentration of technology, to the bond, and to the scheduled appointments.

**CONCLUSIONS:**

The results contribute to comprehending the repeated use of emergency services and provide additional data to plan alternatives to reduce frequent use.

## Introduction

Frequent users are individuals who repeatedly seek emergency services over a given period and may constitute up to 31% of the consultations^(^ 1 ^)^. These users cause an impact on the flow of arrivals, showing a significant contribution to the overloading and overcrowding of these services, as well as on the health system costs^(^ 2 ^)^.

The demand for care in emergency departments is influenced by factors that include social and epidemiological issues, as well as aspects related to the organization of the health system and insufficient structuring of the services^(^ 3 ^-^ 4 ^)^. For many users, these services represent a care alternative and constitute a point of entry to the health system, with the possibility of access to higher technology care of greater resolutivity^(^ 5 ^-^ 6 ^)^. Thus, frequently seeking the emergency services may indicate barriers in the use of the health care network, as well as vulnerability for people who repeatedly need care.

From the perception of health professionals, frequent users present diffuse and undue complaints to the service, which should be resolved in another care level. These users are often stigmatized because their care is considered to be a waste of time and an inappropriate use of the emergency service resources^(^ 7 ^-^ 8 ^)^.

However, it was found that frequent users have poorer health compared to infrequent users. They also present complaints more appropriate for care in emergency services, high prevalence of chronic diseases that lead to increased use, and high rates of hospitalization and mortality^(^ 9 ^-^ 10 ^)^.

These individuals need care in diverse health services, as the isolated use of hospital emergencies services may be insufficient for the resolution of the health needs of the users, due to characteristics of timely and fragmented care^(^ 11 ^-^ 12 ^)^. Without adequate continuous monitoring in the health care network, exacerbations and the use of the emergency services become more frequent, in a cyclic process.

Frequent use is present in the emergency services of various countries such as the United States^(^ 2 ^,^ 7 ^)^, Canada^(^ 1 ^,^ 10 ^)^ and England^(^ 13 ^)^, and has shown steady growth, both in terms of the number of users as well as the number of recurrences^(^ 14 ^)^. It is therefore a focus of interest and concern for healthcare managers^(^ 15 ^)^. However, existing studies are limited to the description of the sociodemographic characteristics, without considering the analysis of the reasons that lead individuals to repeatedly seek care in the emergency services. In Brazil, there are no studies that deal with the theme, demonstrating a gap in the knowledge^(^ 11 ^)^.

The study of this subject can contribute to nursing, providing support for planning alternatives to reduce frequent use. The profile of these individuals and the reasons for the frequent use of emergency services in the national scenario provide useful information to identify preventable factors for the return to the service and to develop care plans that meet the needs of users, qualifying the nursing care.

Given the above, this study aimed to identify the profile of frequent users of emergency services, to verify the associated factors and to analyze the reasons for the frequent use of the services.

## Methodology

A mixed research method was adopted, characterized by a sequential explanatory design, through the development of a quantitative approach phase followed by a qualitative phase^(^ 16 ^)^. Initially a cross-sectional epidemiological study was performed to identify the profile of the frequent users and the associated factors, followed by the qualitative phase that aimed to explore the reasons for repeated use of the service.

The study was conducted in an emergency service of a large hospital in southern Brazil. The individual was considered a frequent user when they sought care four or more times within the period of one year, which is the definition most commonly used among researchers of this theme^(^ 11 ^)^. Thus, the study population included individuals over 18 years of age who used the service four or more times between January and December 2011.

A simple random sample of 385 frequent users (n=385) was defined, using the formula for estimation of proportions, with an acceptable error margin of 5%, confidence level of 95%, and a predicted sample loss rate of 10% . Frequent users were identified from a query of the computerized hospital management system. The sample selection was made from a randomized selection of the users included in the query, conducted using the Statistical Package for the Social Sciences (SPSS) version 18.0. Individuals who received gynecological and surgical care were excluded due to their specific characteristics and low prevalence in the service.

Data from the quantitative stage, referring to the records from January to December 2011, were collected from the electronic medical records of the users between April and May 2012. The variables studied were demographic (age, gender), clinical (number of recurrences and morbidity) and use of the service (day and shift of the care, origin, level of risk classification according to the Manchester protocol used in the service, length of stay in the emergency service, and outcome after the care). It should be noted that the service use variables were collected from the records of the last care received in 2011.

Data were compiled in a Microsoft Excel spreadsheet and transported to the SPSS software, version 18.0. Descriptive statistics were used, with the presentation of data frequency distribution and measures of central tendency, and inferential statistics, with the performance of Fisher's exact test to verify the association between the qualitative variables and of the non-parametric Spearman correlation between quantitative and ordinal variables, with a significance level of 5% (p<0.05) and adjusted residual greater than 1.96. The number of times that the individual sought the service was defined as the dependent variable, and for the performance of Fisher's exact test, the variable was categorized into three groups: Group 1, slightly frequent users (used the service four to six times in the year); Group 2, moderately frequent users (seven to eleven times in the year); and Group 3, highly frequent users (more than twelve times a year).

For the qualitative phase, an intentional sample of 18 subjects was defined, selected based on the results obtained in the quantitative phase, considering the number of recurrences in the service. For the representation of different groups of frequent users seven users from Group 1, seven from Group 2: and four from Group 3 were included.

To collect the qualitative data, semi-structured interview were carried out by telephone, in July and August 2012. Questions addressed the reasons for seeking care in the emergency service, the advantages and disadvantages of using the service and the use of other health services, including those of emergency. To analyze the data of this phase, thematic content analysis^(^ 17 ^)^ was used, operationalized by the Atlasti software.* 6*.

The development of the study met national and international human research ethics standards.

## Results

In 2011, 24,912 individuals sought care in the emergency service, with 2,187 being (8.8%) frequent users. These users required 12,075consultations, which corresponded to 24.5% of the total. The number of recurrences ranged from 4 to 58, with the mean being 6.59 and standard deviation of 4.19. It was found that 251 (65.2%) were members of Group 1 (slightly frequent), 117 (30.4%) of Group 2 (moderately frequent) and 17 (4.4%) of Group 3 (highly frequent).

Patients were predominantly female (54.8%), elderly people (42.9%) and presented chronic diseases (84.9%). The mean age was 53.37 years (standard deviation=18.26). The majority of the individuals sought the service due to spontaneous demand (85.4%), on weekdays (81.3%) and during the morning shift (59.1%). A total of 27% of the users were directed by the emergency professionals to return for re-evaluation.

According to the risk classification used in the service (Manchester Protocol), the majority of the users (63.5%) were classified in the highest category (emergency, very urgent and urgent). With regard to the length of stay, 42.1% remained in the emergency service for more than 24 hours.

Regarding the care outcome, 46.5% were discharged, 23% were referred for hospitalization and 3.9% died while in the service. Some individuals, after the screening, were sent to the outpatient department of the hospital or to the primary care unit (21.5%), not staying in the emergency service to receive care.

The scheduled follow-up appointment, length of stay and outcome variables showed significant differences, as presented in Table 1. Adjusted residual analysis showed that users of Group 1 were associated with scheduled appointments in the service, with a stay of up to one hour in the unit and with referrals for outpatient care elsewhere. Users of Group 2 were associated with a stay of one to 12 hours in the service and with hospital discharge. Users of Group 3 presented no significant difference, however, tendencies of 12 to 24 hour stays in the emergency service and death were identified, with values close to statistical significance.

**Table 1 -:** Comparison between groups of frequent users, according to the qualitative variables. Porto Alegre, RS, Brazil, 2012

Variable		Sample* (N=385)	Frequent user group			p^†^
			Group 1* (n=251)	Group 2* (n=117)	Group 3* (n=17)	
		N (%)	n (%)	n (%)	n (%)	
Gender						0.588
	Female	211 (54.8)	135 (53.8)	68 (58.1)	8 (47.1)	
	Male	174 (45.2)	116 (46.2)	49 (41.9)	9 (52.9)	
Age in years						0.080
	18 – 40	101 (26.2)	68 (27.1)	31 (26.5)	2 (11.8)	
	41 – 59	119 (30.9)	84 (33.4)	31 (26.5)	4 (23.5)	
	60 – 79	142 (36.9)	81 (32.3)	50 (42.7)	11 (64.7)	
	> 80	23 (6.0)	18 (7.2)	5 (4.3)	0	
Chronic diseases						0.870
	Yes	326 (84.9)	212 (84.5)	100 (86.2)	14 (82.4)	
	No	58 (15.1)	39 (15.5)	16 (13.8)	3 (17.6)	
Origin						0.576
	Spontaneous demand	328 (85.4)	213 (84.9)	100 (86.2)	15 (88.2)	
	Ambulance	9 (2.4)	7 (2.8)	2 (1.7)	0	
	Primary healthcare	2 (0.5)	1 (0.4)	0	1 (5.9)	
	Outpatient clinic	42 (10.9)	28 (11.2)	13 (11.2)	1 (5.9)	
	Other	3 (0.8)	2 (0.8)	1 (0.9)	0	
Scheduled appointment						0.011
	Yes	104 (27)	80 (31.9)^ ‡^	22 (18.8)	2 (11.8)	
	No	281 (73)	171 (68.1)	95 (81.2)	15 (88.2)	
Days of the week						0.206
	Weekdays	313 (81.3)	207 (82.5)	95 (81.9)	11 (64.7)	
	Weekend	71 (18.7)	44 (17.5)	21 (18.1)	6 (35.3)	
Shift						0.407
	Morning	225 (59.1)	155 (62.2)	60 (52.2)	10 (58.8)	
	Afternoon	88 (23.1)	51 (20.5)	33 (28.7)	4 (23.5)	
	Night	68 (17.8)	43 (17.3)	22 (19.1)	3 (17.6)	
Risk classification						0.470
	Emergency	24 (6.9)	16 (7.0)	6 (5.5)	2 (15.4)	
	Very urgent	63 (18.0)	36 (15.8)	26 (23.9)	1 (7.7)	
	Urgent	135 (38.6)	86 (37.7)	44 (40.4)	5 (38.5)	
	Slightly urgent	121 (34.6)	85 (37.3)	31 (28.4)	5 (38.5)	
	Not urgent	7 (2.0)	5 (2.2)	2 (1.8)	0	
Length of stay in hours						0.007
	< 1	94 (24.4)	76 (30.3)^ ‡^	15 (12.8)	3 (17.6)	
	1 – 12	128 (33.3)	71 (28.3)	51 (43.6)^ ‡^	6 (35.3)	
	12 – 24	38 (9.9)	22 (8.8)	13 (11.1)	3 (17.6)	
	24 – 48	39 (10.1)	21 (8.3)	16 (13.7)	2 (11.8)	
	> 48 hours	86 (22.3)	61 (24.3)	22 (18.8)	3 (17.6)	
Outcome						0.001
	Discharged	179 (46.5)	106 (42.4)	67 (57.8)^ ‡^	6 (35.3)	
	Referral to outpatient care	83 (21.5)	70 (28.0)^ ‡^	10 (8.6)	3 (17.6)	
	Hospitalization	88 (23)	54 (21.6)	29 (25.0)	5 (29.4)	
	Death	15 (3.9)	10 (4.0)	3 (2.6)	2 (11.8)	
	Left the service	11 (3.4)	7 (2.8)	4 (3.4)	0	
	Other	7 (1.8)	3 (1.2)	3 (2.6)	1 (5.9)	

* Percentages calculated excluding variables without response

† Using Fisher's exact test

‡ Statistically significant association from the adjusted residual tests

Table 2 shows that there was a weak, although significant, inverse correlation between the variables risk classification and user recurrence in service. There was also a weak positive correlation between length of stay and number of recurrences. The data suggest that the more times the individual sought care, the more the individual was classified in the higher risk categories, considered urgent, and stayed longer in the emergency service.

**Table 2 -:** Correlation between number of recurrences in the service and quantitative and ordinal variables. Porto Alegre, RS, Brazil, 2012

	Recurrences in the service	
	Correlation coefficient (r)	p*
Age	+0.067	0.191
Risk classification	-0.106	0.048
Length of stay	+0.107	0.035

* Using Spearman's correlation test

**Figure 1 -:**
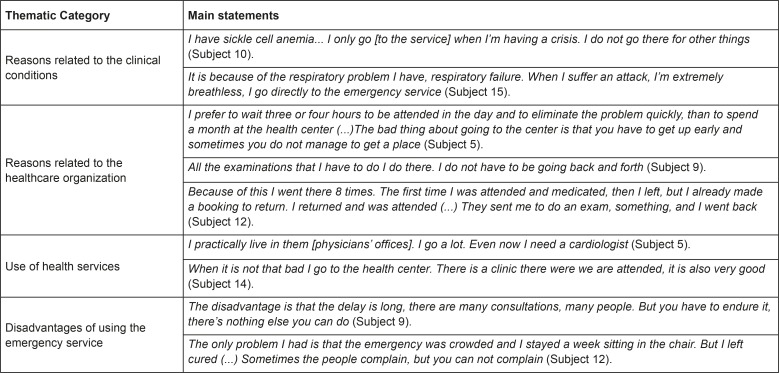
Description of the thematic categories related to the reasons for the frequent use of the emergency service. Porto Alegre, RS, Brazil, 2012

Among those interviewed, half were male, seven were aged between 18 and 40 years, six between 41 and 60 years, and five aged more than 61 years. According to the classification levels of the Manchester protocol, two were classified for immediate care, three with high risk, five with moderate risk, six with low risk, one as not urgent and one had no record of the risk classification in the medical records. The empirical material obtained in the interviews was grouped into four thematic categories, as shown in Figure 1.

It was found that the majority of the respondents sought care in the emergency service due to the exacerbation of chronic diseases, such as hypertensive, asthma and hypoglycemic crises. Recurring acute injuries or those that require prolonged treatment were also mentioned. For the individuals, their situations were urgent and they needed immediate care.

Reasons related to the healthcare organization in the country influenced the choice of the service to be used. The ease of access to emergency units, compared to those of other health services (especially primary care), the valorization of technology concentrated in one place, the perception of resolvability of health problems from previous experiences, and the bond with the professionals and the hospital are some of the aspects that motivated users to seek the hospital emergency service.

Another reason that contributed to the frequent use was the follow-up appointment requested by the health team. When users were discharged from the emergency service and the professionals evaluated the need for follow-up treatment, they were asked to come back for re-evaluation of the health status, which led to non-urgent demands due to stabilized health situations.

It was identified that the users used primary care units and the outpatient clinic of the hospital continuously, through programmed actions. Some individuals highlighted seeking care in the referral units for situations of low severity and urgency, opting for the hospital emergency service for situations requiring immediate care.

Despite the emergency service presenting various advantages in their use, some negative points were referred to by the individuals, including overcrowding, high demand, delays to receive care and inadequate facilities. However, they mentioned that the advantages overcome the disadvantages, as the users submit themselves to the discomfort for the purpose of obtaining care.

## Discussion

Frequent users accounted for a small percentage of the total number of service users, however, were responsible for a considerable demand for care. A recent study showed a similar prevalence of frequent users to that found^(^ 2 ^)^, while others demonstrate higher values^(^ 1 ^,^ 15 ^)^, showing the impact of repeated use on the emergency points of entry.

The predominance of the female gender, older age group, and chronic diseases in the profile of the frequent users is similar to that described in studies in the international context^(^ 9 ^,^ 12 ^,^ 18 ^)^. It should be noted, however, that both the mean age and the prevalence of chronic diseases were higher in this study than in others^(^ 10 ^,^ 12 ^,^ 19 ^)^.

The high prevalence of elderly people and those with chronic diseases may indicate the potential vulnerability of these individuals to require more healthcare, so that, in many circumstances, the use of the emergency service is necessary and appropriate, while in others it is the result of unresolved health needs that culminate in exacerbations^(^ 20 ^)^. Although in this study there were no significant differences found between the frequent user groups, authors state that the age and presence of chronic diseases are factors associated with the repeated use of emergency services^(^ 12 ^,^ 19 ^)^.

The findings of association and correlation of the risk classification and the length of stay with frequent use progression are consistent with results of studies that identified that the proportion of urgent cases grew with the increase of recurrences in the service^(^ 13 ^)^ and that frequent users stayed in the service longer than the infrequent users^(^ 10 ^)^.

In this study no associations were identified between the hospital outcome or death and frequent users, however, it was shown that the hospitalization rate was 23%, higher than the 8.8% found in a study on the general use of emergency services in southern Brazil^(^ 21 ^)^. However, the literature highlights that frequent users have increased chances of hospitalization or death after emergency room treatment than infrequent users^(^ 9 ^-^ 10 ^)^.

The results corroborate the fact that frequent users are people that are more ill than the general population of the emergency services, due to the characteristics of high rates of chronic diseases, of urgent risk classification, of mortality, of hospitalization and longer stays in the service^(^ 7 ^,^ 18 ^)^.

However, the reasons for the frequent use indicate that the emergency services have become alternatives for care. Aspects related to ease of access, to the perception of greater resolvability and technology, to the formation of a bond and to scheduled appointments are some of the reasons that correspond to factors extrinsic to the individuals that contribute to the frequent use. These factors have been reported in the literature^(^ 5 ^-^ 6 ^)^ which, although not dealing exclusively with frequent users, demonstrate the relevance of the discussion regarding weaknesses in the organization of the healthcare network in an attempt to reduce seeking care in emergency services.

It should be noted that, unlike the data found in studies of the general use of emergency services^(^ 21 ^-^ 22 ^)^, the restricted hours of the outpatient services did not seem to be a major cause for frequent demand. The days and times when other services are open were those most sought by frequent users, with no subject reporting having had problems accessing health services due to opening hours.

The findings related to scheduled appointments illustrate the concern of professionals with the continuity of the care initiated in the emergency service, and the lack of articulation between the emergency service and the other services of the healthcare network. It is believed that there are difficulties regarding referring users to primary and specialized healthcare, causing the emergency professionals to prefer to maintain the bond to the institution than leave them to search around for care. This, however, indicates a distortion of the purpose of the service^(^ 6 ^)^. The recognition by the emergency service professionals of the impact of this measure is important for reflection and a change of practices.

Contrary to the perception of health professionals that emergency services are the only source of care for frequent users, the use of complementary services of primary and specialized care was identified, which is consistent with results from studies performed in Canada^(^ 1 ^)^ and the United States^(^ 7 ^,^ 15 ^)^. However, it was shown that users seek the emergency service to receive rapid service, which is not always possible to obtain in the primary and specialized care, which organize their care as a programmed schedule, with restricted space for spontaneous demands, causing dissatisfaction for the users^(^ 23 ^)^.

Thus, while negative points were assigned to the use of the emergency service, such as long waiting times for care and inadequate facilities, the image of these services is more favorable than that of the others^(^ 24 ^)^ due to guaranteeing care.

Despite the relevance of the results, limitations must be considered due to the complexity of the phenomenon and the methodological design. The use of only one hospital emergency service restricted the data analysis and generalization of the results, considering that frequent users could use more than one emergency service. The use, albeit cautious and controlled, of data collected from the electronic medical records can be a limitation, as this is information provided by the professionals during the care and it is not possible to know if the records were complete.

Despite these limitations, the main findings of this study provide important contributions for the comprehension of the repeated use of emergency services, as well as offer support for planning interventions with frequent users. Efforts are needed to identify individuals who are at risk of becoming frequent users, in order to take actions to prevent recurrences. Case management and the development of care plans are strategies that ensure the referral and adequate transition of users and care between the health system services, providing continuity of care for the frequent users within the healthcare network. Such interventions have been shown to be useful for the reduction of repeatedly seeking care in emergency services, for the reduction of costs and for the improvement of the clinical and social conditions of individuals^(^ 25 ^)^. Accordingly, making the counter-referral effective in emergency services and the nursing team's performance in the care transition process are essential to organize the flow of use of health services and to reduce the frequent demand for hospital emergency services.

## Conclusion

The profile of frequent users of the emergency service presented some predominant characteristics, such as females, elderly people, sufferers of chronic conditions, seeking care due to spontaneous demand on weekdays and during the day, urgent risk classifications, long stays in the service and high hospitalization rates.

Slightly frequent users were associated with scheduled follow-up appointments in the service, with a stay of up to one hour in the unit and with referrals for outpatient care elsewhere. Moderately frequent users were associated with a stay of one to 12 hours in the service and with hospital discharge. In turn, highly frequent users showed a tendency for longer stays in the emergency service and for death. The risk classification and the length of stay were correlated with the number of recurrences of the user in the service.

Clinical reasons to seek care in the emergency service were evidenced, predominantly the exacerbation of chronic diseases, as well as reasons related to the health system organization, such as ease of access, perception of greater resolvability and the provision of technology, the formation of bonds and scheduled follow-up appointments. The users used primary and specialized care continuously, however, the hospital emergency service was considered an alternative for rapid care.

The frequent use of emergency services should be included in the agenda of research in public health, due to its relevance in the national and international context.
